# Machine Learning Based Biomimetic Underwater Covert Acoustic Communication Method Using Dolphin Whistle Contours

**DOI:** 10.3390/s20216166

**Published:** 2020-10-29

**Authors:** Jongmin Ahn, Hojun Lee, Yongcheol Kim, Wanjin Kim, Jaehak Chung

**Affiliations:** 1 Department of Electrical and Computer Engineering, INHA University, Incheon 22212, Korea; anjong3@naver.com (J.A.); timmit@naver.com (H.L.); dydcjf4691@naver.com (Y.K.); 2 Agency of Defense Development, Jinhae-gu 51682, Korea; kimwj@add.re.kr

**Keywords:** secure LPD/LPI communication, modulation, signal processing, bio-mimetic

## Abstract

For underwater acoustic covert communications, biomimetic covert communications have been developed using dolphin whistles. The conventional biomimetic covert communication methods transmit slightly different signal patterns from real dolphin whistles, which results in a low degree of mimic (DoM). In this paper, we propose a novel biomimetic communication method that preserves the large DoM with a low bit error rate (BER). For the transmission, the proposed method utilizes the various contours of real dolphin whistles with the link information among consecutive whistles, and the proposed receiver uses machine learning based whistle detectors with the aid of the link information. Computer simulations and practical ocean experiments were executed to demonstrate the better BER performance of the proposed method. Ocean experiments demonstrate that the BER of the proposed method was 0.002, while the BER of the conventional Deep Neural Network (DNN) based detector showed 0.36.

## 1. Introduction

For military underwater acoustic (UWA) communication systems, low probabilities of detection/intercept (LPD/LPI) are important parameters [1,2,3,4,5,6,7]. In general, since the energy of the received signals is measured for detecting the existence of an enemy, covert communications have been developed to reduce the power spectral density of the communication signal that spreads the transmission energy over a wide bandwidth to make it appear similar to background noise [1,2,3,4,5,6,7]. In the UWA communications, however, the available bandwidth is too small to spread the signal energy [1,2,3,4,5,6,7]. Even though low energy transmission methods with narrow bandwidths have been developed, these schemes suffer from a large bit error rate (BER) [1,2,3,4,5,6,7].

As an alternative, biomimetic covert UWA communications which mimic the biological sounds of underwater animals have been developed to overcome the problem of the conventional covert communications [8,9,10,11,12,13,14,15]. The mimicked bio-sounds enable the enemy to confuse the communication signals with the underwater animal sounds. Since the mimicked sound does not need to reduce the transmission energy, a better BER performance can be achieved than that of the conventional covert communications [8,9,10,11,12,13,14,15]. Thus, the biomimetic covert communication schemes for the UWA communications have been considered as one of emerging covert communications and dolphin whistles have been utilized for the covert underwater animal sounds [8,9,10,11,12,13,14,15]. Many bio-mimetic communication schemes are listed in Table 1.

Bio-mimetic communication schemes have been researched since 2013. The authors in [8] developed the Pulse Position Modulation (PPM) based dolphin whistles. This method was tested in river experiments and its BER performance was not analyzed by the computer simulations. Only selected whistles were tested to obtain better BER results. In [9], the phase shift keying (PSK) modulation with dolphin whistles was utilized, but BER at an Signal to Noise Ratio (SNR) range of 5 dB to 10 dB showed 10^−2^, which was inappropriate for communication, and the scheme was not tested in ocean experiments. Chirp spread spectrum (CSS), frequency shift keying (FSK) and PSK were utilized based on the dolphin whistle contour [10,11,12,13]. However, the schemes in [10,11,12,13] distorted dolphin whistles due to allocating binary information to the whistles, and had low covertness. Even though the methods in [14,15] were developed to utilize dolphin whistles without distorting the whistles, the algorithm in [14] showed a relatively large BER performance (10^−2^) at an SNR range of 5 dB to 10 dB, and the scheme in [15] had to utilize only high auto-correlated whistles for modulation, which decreased the covertness. Thus, a low BER performance in the ocean experiments and covertness, i.e., degree of mimic (DoM), are the most important issues of the biomimetic communication scheme.

In this paper, we propose a biomimetic covert communication scheme that modulates the information bits into various whistle patterns to increase the DoM with the link information among consecutive transmitted whistles, and detects the distorted whistles—via the link information—with the UWA channel using a machine learning based detector. The proposed method divides a large number of dolphin whistles into groups based on the similarity of the patterns. Each group is used as a symbol and mapped to information bits. To convey information bits and maximize the DoM, the randomly selected whistle in a chosen group is transmitted, and different whistles are sequentially transmitted. When a number of transmitted whistles pass through the UWA channel and background noise is added, the conventional machine learning based detectors suffer from detecting many distorted whistles. However, the proposed scheme, that utilizes a multi-stage directional acyclic graph (DAG)-net and a long-short term memory (LSTM), attains a low BER and large DoM.

The main contributions of the proposed method are as follows,

- For a large the DoM, we directly utilize many real dolphin whistles for the modulation.
- For a small BER, we optimally classify the real dolphin whistles with large distances, and develop a trellis structured transmission algorithm using the information link matrix, without sacrificing the DoM.
- For increasing the detection performance of the nonlinear characteristics of many transmitted whistles, we develop a DAG-net based machine learning detector.
- For increasing the BER performance, we combine the DAG-net and LSTM, i.e., D-LSTM, which utilizes the link information to demodulate the distorted whistles received by the UWA channels.
- The performance of the proposed algorithm has been proved through computer simulations and practical ocean experiments.

The paper is organized as follows. Section 2 describes the characteristics of the dolphin whistles and the whistle classification by groups. Section 3 proposes the modulation method that allocates bit information into whistles using the link information. Section 4 mentions the DAG-net based LSTM demodulator. In Section 5, the learning process of the proposed method is shown. Section 6 demonstrates the BER performance by using computer simulations and practical ocean experiments. Section 7 concludes the paper.

## 2. Whistle Classification

**Figure 1:**
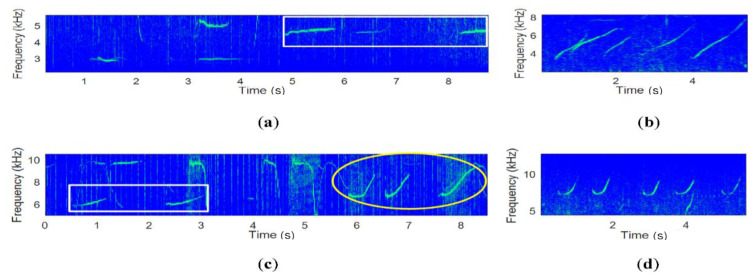
The spectrograms of the false killer whale whistles. (**a**) Flat shape whistle, (**b**) Up chirp whistle, (**c**,**d**) Curved whistle.

**Figure 2:**
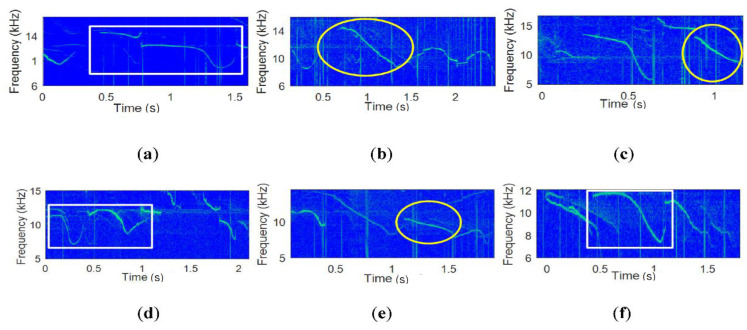
The spectrograms of the white sided dolphin whistles. (**a**,**c**,**d**,**f**) Scop shape whistle, (**b**,**e**) Down chirp whistle.

Dolphins communicate with each other using whistles. The general dolphin whistles have a time duration that varies from several hundred milliseconds to two seconds, and a frequency bandwidth that varies from several hundred Hz to tens of kHz [16,17,18,19,20]. The variation of frequencies over the time duration is referred to as the frequency contour or whistle pattern [16,17,18,19,20]. In Figure 1 and Figure 2, the whistle spectrograms of the false killer whales and white sided dolphins, respectively, are displayed. The frequency components of the dolphin whistles vary in time. In Figure 1 and Figure 2, many dolphins generate many different or similar whistle patterns.

In Figure 1 and Figure 2, the similar whistle patterns are marked by rectangular and circular boxes. In Figure 1, the rectangular and circular boxes contain up-chirps with a different frequency variation and a large variation, respectively. In Figure 2, the rectangular and the circle boxes are marked for flat-downward scoops and down chirps, respectively. In practice, more whistle patterns than in the above examples can be found. If the whistles with similar patterns are classified as the same group and binary bits are allocated to the whistle group, we can transmit binary information with the same dolphin whistle patterns. Thus, the proposed method transmits one of the randomly selected whistle patterns in the group, which preserves a larger DoM and greater covertness than the conventional biomimetic UWA communication methods.

When all whistles are classified as groups, the distance between groups needs to be kept as far as possible to attain the low BER. Thus, we classify the whistles to maximize the distance based on the whistle features.

For whistle classification with the maximum distance, firstly, we set a whistle feature vector (V) whose elements present the dominant features of the whistle, e.g., *L* frequencies of the whistle, whistle duration, maximum and minimum frequencies, chirp rate, etc., and secondly, we maximize the distances based on the vector(V). V is set as below,

(1) $$V=[f(\tau_{1}),\cdot \cdot \cdot ,f(\tau_{L}),\cdot \cdot \cdot ,\max f(t),\min f(t),\cdot \cdot \cdot ]$$

If V consists of the j elements, V has a vector space of $R^{j}$ and the classification with the maximum distance is performed in $R^{j}$ space.

For the classification, we change the classification problem of maximizing distances among different whistle groups, to a new problem of minimizing the distances in the same groups. Since the k-means clustering algorithm is known as a good classification method for minimum variance [19,20], the k-means clustering algorithm is utilized for classifying the whistles as groups.

Let $K_{\mathrm{opt}}$ be the number of groups, in which the variance of whistles in the same group is minimum, and the set of classified groups be $G=(G_{1},G_{2},\cdot \cdot \cdot ,G_{k},\cdot \cdot \cdot ,G_{K_{opt}})$. Assume that the N whistle vector and the average vectors of the k-th group $G_{k}$ is $\mu_{k}$. Then, the k-means algorithm for classifying the whistles is written as [21,22],

(2) $$\underset{G,K_{\mathrm{opt}}}{\arg\min}\sum_{k=1}^{K_{\mathrm{opt}}}\sum_{V_{n}\in G_{k}}|V_{n}-\mu_{k}{|}^{2}$$

Equation (2) minimizes the variance of the vectors ($V_{n}$) which belongs to the same group, i.e., the distance among groups is maximized. Then, $K_{\mathrm{opt}}$ groups are obtained, and the whistles in the same group have similar patterns.

For the biomimetic UWA communication, the information bits are allocated to $G=(G_{1},G_{2},\cdot \cdot \cdot ,G_{k},\cdot \cdot \cdot ,G_{K_{opt}})$ and randomly selected whistles in the group are transmitted. When a large number of information bits are inputted and all whistles are transmitted, the large DoM is attained. At the receiver, the conventional maximum likelihood (ML) based detector can be used to estimate the group index (k) from the received whistles. The ML based detector determines the transmitted bits by extracting the feature vector (${\hat{V}}_{n}$) in Equation (1) and comparing all feature vectors of the whistles ($V_{n}$), finding the closest one.

When the conventional ML detector detects the received whistles, the ML detector suffers from two problems: for the first problem, when the number of real whistles is large, a low BER is not obtained. The maximum distance by the k-means algorithm between groups may not be large enough to overcome the background noise and the UWA channel distortions. If some whistles in the group are picked to keep the larger distance and the error correction schemes are used, the BER performance can increase. However, the DoM and the data rate decreases. Thus, we need to develop a communications method without scarifying the DoM and the BER. For the second, when the received whistles are distorted by the UWA channel, the distorted whistles cannot be compensated by the equalizer. Even though the channel is estimated by the pilots, the frequency bandwidth and time duration of the whistles are larger than the coherent frequency and the coherent time, respectively. In addition, the repeatedly transmitted pilots reduce the DoM.

The small distance problem may be resolved by the modern machine learning detection scheme, whose detection capability is better than the ML. When the whistle patterns are represented as a 2-D image, e.g., spectrogram, the machine learning technique easily learns the overall contours and the detail features of the whistles. However, the distorted whistle problem of the UWA channels may not be overcome by the conventional machine learning methods. This is because the detection accuracy of the conventional machine learning methods may not be enough to satisfy the common BER requirements of the communications.

Therefore, we propose a biomimetic communication scheme: the transmitter modulates the whistles with the larger distances and DoM based on the link information among adjacent whistles, and the receiver demodulates and detects the distorted whistles using a DAG-net based LSTM with additional link information among whistles. The proposed modulation and demodulation method are described in the following sections.

## 3. Biomimetic Covert Whistle Transmitter

For a low BER, the conventional digital communication utilizes the forward error correction (FEC) method. The FEC provides a connection rule to concatenated additional symbols and corrects the erroneous bits, but requires the additional bits that reduce the data rate [23,24]. In cases of small bandwidth UWA communication systems, the data rate is one of the important parameters. Thus, the proposed biomimetic communication scheme is developed to obtain a low BER and large DoM, without sacrificing the data rate. The proposed method reclassifies the real whistles into a large number of subgroups to have larger distances among subgroups and provide link information among subgroups to utilize all subgroups. The detailed procedure and an example are described in this section.

Assume that the maximum number of groups for the optimum accuracy is given as $K_{\mathrm{opt}}$, and the maximum number of bits per whistle is given as $M_{\mathrm{opt}}=\left\lfloor \log_{2}K_{\mathrm{opt}}\right\rfloor$, and $K_{\mathrm{opt}}$ may not be the same as $2^{M_{\mathrm{opt}}}$. To increase the BER performance and preserve the same data rate, the proposed method reclassifies the $K_{\mathrm{opt}}$ groups into enlarged subgroups $K_{\mathrm{tot}}=(2^{\alpha }\times K_{\mathrm{opt}})$, and generates many subgroup sets, in which one subgroup set consists of $2^{M_{\mathrm{opt}}}$ subgroups among $K_{\mathrm{tot}}$ subgroups. The possible number of the subgroup sets with the size of $2^{M_{\mathrm{opt}}}$ is very large, but in this paper, $K_{\mathrm{tot}}$ subgroup sets that have large distances are chosen. If we carefully select the subgroup sets whose distances are larger than those of the first classified $K_{\mathrm{opt}}$ group sets, we attain a lower error rate performance.

The proposed link information that connects all subgroup sets is determined by the link matrix (H) with the size of $K_{\mathrm{tot}}\times 2^{M_{\mathrm{opt}}}$ that one row consists of the one subgroup set with a size of $2^{M_{\mathrm{opt}}}$ and the total number of rows is $K_{\mathrm{tot}}$. The i-th column index is mapped to input bits for the binary allocation, e.g., binary counter or Gray code, and every element value of H indicates the current subgroup and the row number for the next input bits. Then, the link information between two subgroup sets is established. In order to avoid falling into a short loop by the subgroup loop indexing, the element value of a row of H is not allowed to include the same row index and the elements of the one subgroup set is not the same as that of other subgroup sets, and all indices occur evenly $2^{M_{\mathrm{opt}}}$ times. This additional link information helps the receiver to decode the distorted received symbols and attain a better BER performance. A link matrix H is given as below,

(3) $$H=\left[\begin{array}{ccccc} h_{1,1} & \cdots & h_{1,i} & \cdots & h_{1,2^{M_{opt}}}\\ & & \vdots & & \\ h_{k,1} & \cdots & h_{k,i} & \cdots & h_{k,2^{M_{opt}}}\\ & & \vdots & & \\ h_{K_{tot},1} & \cdots & h_{K_{tot},i} & \cdots & h_{K_{tot},2^{M_{opt}}} \end{array}\right]$$

Assume that a N bit stream is represented as the vector of $B=\{b_{1},\cdot \cdot \cdot ,b_{n},\cdot \cdot \cdot ,b_{N}\}$, where the size of $b_{n}$ is equal to $M_{\mathrm{opt}}$. When $b_{1}$ is input, the $b_{1}$ binary value is translated to a decimal number which is the index of the column of the first row. The selected element of the first row indicates the subgroup index for $b_{1}$, and a whistle in the indicated subgroup is randomly chosen and transmitted. Subsequently, the element selected by $b_{1}$ also provides the row number of H for the next input $b_{2}$. For $b_{2}$, the same procedure is performed for the input $b_{1}$. This procedure is executed to all elements of B. If the size of B is large, all elements of H will be picked and all whistles will be utilized for the transmission, which preserves the large DoM.

**Figure 3:**
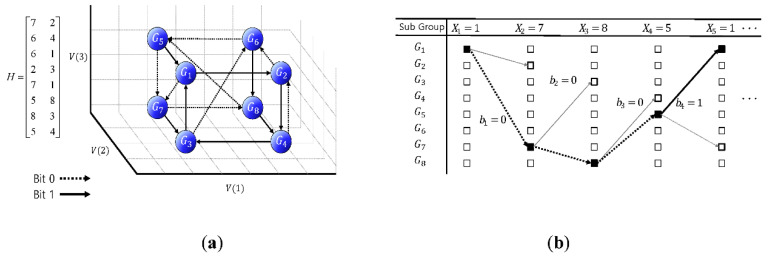
(**a**) Link between adjacent whistle subgroups; (**b**) sequential link by H with input 0001.

As an example, Figure 3a shows the H matrix for α=2, $K_{\mathrm{opt}}=2$, $M_{\mathrm{opt}}=1$, $K_{\mathrm{tot}}=8$, and the links, and Figure 3b displays the sequential link connections.

In Figure 3a, the first and the second columns of H are mapped to “0” or ”1”, respectively, of input bits. No same subgroup set, e.g., (7,2), (6,4), etc., is observed and the subgroup numbers are randomly distributed and appear two times, i.e., $2^{M_{\mathrm{opt}}}$. Let the n-th transmitted whistle be $W_{n}$, and the subgroup index of the whistle be $X_{n}$. If the input bit stream is 0001 and the first bit starts with the first row, the first bit “0” is mapped to the first column of the first row in H. The first column of the first row of H reads “7”. One whistle ($W_{1}$) in the seventh subgroup ($X_{7}$) is randomly selected and transmitted as the first symbol. For the next bit, the “7” also indicates the next row number of H. For the second bit “0”, the first column of the seventh row reads “8”. One whistle ($W_{2}$) is randomly selected from the eighth subgroup ($X_{8}$) and transmitted for the second symbol, and “8” also indicates the eighth row for the third input bit. Similarly, for the fourth bit “1”, the subgroup selection is performed in the fifth row which is chosen by the third bit. Since the input bit is 1, the second column of the fifth row is selected and reads “1”. One whistle ($W_{4}$) of the first subgroup ($X_{1}$) is randomly picked and the first row is chosen for the fifth input bit. This sequential link result is depicted in Figure 3b.

Therefore, the proposed modulation method utilizes all whistles that obtain a large DoM, and provides the large distance among the subgroup sets and the additional link information, which attains the low BER. For the demodulation using the link information, a machine learning based sequential detection is proposed since the machine learning detection outperforms the conventional feature detection methods and the sequential link information also enhances the detection performance. The detailed demodulation scheme is described in the next section.

## 4. Machine Learning Based Mimetic Whistle Receiver

The conventional machine learning detector may not obtain a great enough BER performance for the communications. Thus, a novel biomimetic demodulation method is proposed that detects the received bits from the distorted whistles by the UWA channel, using the additional link information. Please note that the proposed method does not estimate the UWA channels.

The transmitted biomimetic N whistles ($W=\{W_{1},W_{2},\ldots ,W_{n},\ldots ,W_{N}\}$) go through the UWA channel (H) and reach to the receiver sensor, and the noise ($N=\{N_{1},N_{2},\ldots ,N_{n},\ldots ,N_{N}\}$) is added. The received whistle ($Y=\{Y_{1},Y_{2},\ldots ,Y_{n},\ldots ,Y_{N}\}$) is modeled as,

(4) Y=H∗W+N

where ∗ denotes a convolutional operation and all parameters are composed of vectors. As in Section 3, the *n*-th element ($W_{n}$) of the transmitted whistle vector (W) represents randomly selected whistles chosen from the *n* -th subgroup ($X_{n}$) in the vector X.

The purpose of the proposed method is to estimate the transmitted group index $\hat{X}$ from the received whistle Y using the additional link information among sequential whistles. This estimation can be performed by finding a maximum probability of $\hat{X}$ for Y, and expressed as,

(5) $$\hat{X}=\underset{{\hat{X}}_{1},\cdots ,{\hat{X}}_{N}}{\max}p\left(Y_{1}\in {\hat{X}}_{1},\cdots ,Y_{n}\in {\hat{X}}_{n},\cdots ,Y_{N}\in {\hat{X}}_{N}\right)$$

In Equation (5), in general, maximum likelihood sequence detection (MLSD) can be performed for the link information to increase the estimation performance of $\hat{X}$. However, when the transmitted whistles pass through the UWA channel, the received whistles are severely distorted by the UWA channel, and the maximum likelihood (ML) detector itself does not have a great enough detection performance. Thus, the MLSD does not provide a useful detection performance, despite the additional link information [23,24].

In order to achieve the large BER performance, the detection performance of the whistle itself needs to be improved, and then, the link information among whistles is jointly utilized. In Figure 1 and Figure 2, the whistles have a nonlinear time-frequency change property, and are heavily distorted by the UWA channel. Thus, this paper develops a sophisticated machine learning detection method for attaining the large detection performance and the LSTM for utilizing the link information among whistles.

### 4.1. Machine Learning Network Structure for Biomimetic Receiver

**Figure 4:**
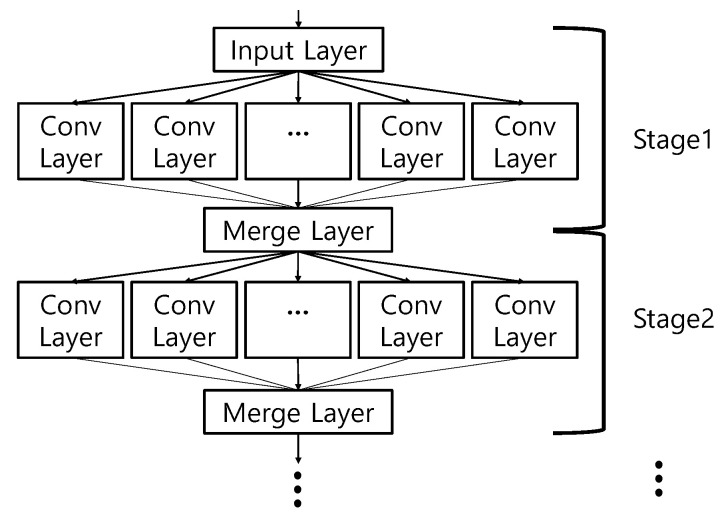
Directional acyclic graph (DAG)-net structure.

The proposed machine learning network is designed to extract the features of whistle subgroups from the received whistles with the link information. For the training data, two test sets with classified real dolphin whistles are made: one set is obtained by only adding the noise, and the other set is made by passing the UWA channel and adding the noise. The test whistles are transformed into the 2-D time-frequency plane using the spectrogram. In this paper, the DAG-net that is composed of the parallel convolutional layers (CLs) and the merging layers is proposed, and is shown in Figure 4.

In Figure 4, the parallel CLs of the DAG-net are constructed to simultaneously extract the nonlinear contours and the detail whistle features from the input spectrogram. The extracted features are merged and transferred to the next stage [25,26,27]. The final stage extracts the detailed features and estimates the subgroups. The parallel CL structure of the DAG-net shows a better feature extraction performance than the conventional Deep Neural Network (DNN) in the simulation section.

**Figure 5:**
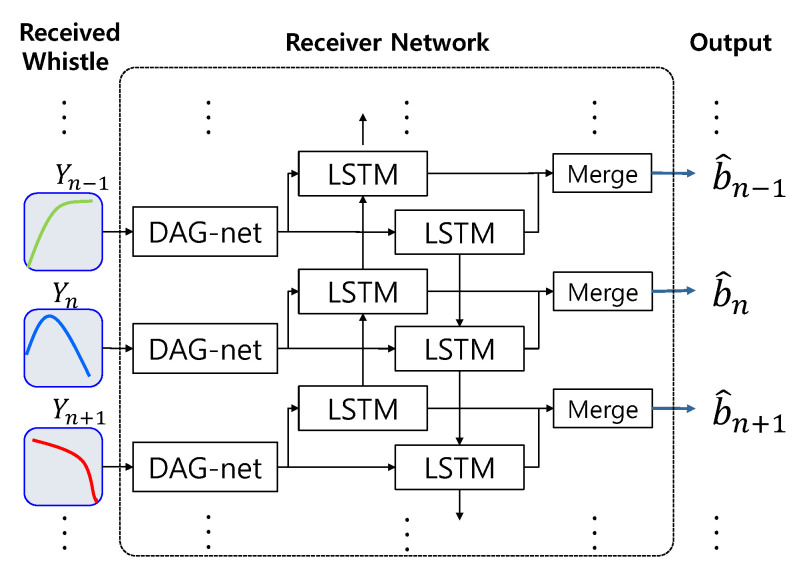
DAG-net and LSTM (D-LSTM) network structure. LSTM: long-short term memory.

The misdetection performance of the designed DAG-net for the distorted whistles shows approximately a 10^−2^ order, which is better than that of the ML detector and may be enough for some applications. However, this detection performance may not be acceptable to the communication systems [28,29,30,31,32]. In this case, the link information plays an important role in increasing the detection performance, and the LSTM utilizes the link information. Among many LSTMs, Bi-directional Long Short Term Memory (Bi-LSTM) stores the input information in both long and short term memories and effectively analyzes the link information back-and-forth [33,34]. Thus, we design the D-LSTM that consists of the DAG-net and the Bi-LSTM, in which the DAG-net is for extracting the features of whistles and the Bi-LSTM is for utilizing the link information between whistles. The proposed D-LSTM method shows a lower BER than the DAG-net without the link information. The proposed D-LSTM structure is shown in Figure 5.

In Figure 5, the spectrogram sequence of Y is input and the estimated bits are output. The analyzed features of the whistles by the DAG-net are transferred to the Bi-LSTM, which extracts the link information and demodulates received bits. The following section describes the training and detection methods of the proposed D-LSTM.

### 4.2. D-LSTM Training Method and Biomimetic Receiver

**Figure 6:**
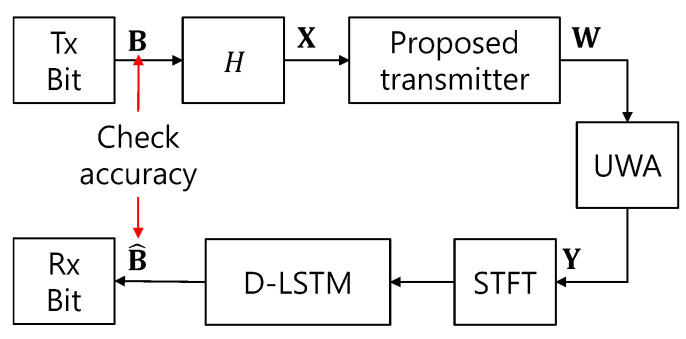
D-LSTM network training strategy. UWA: underwater acoustic.

The spectrogram of Y in Equation (4) is obtained from taking Short Time Fourier Transform (STFT) and is used for the input training data of the machine learning networks. The proposed D-LSTM network learns the spectrogram of Y to check the accuracy between the input bits (B) and the estimated bits ($\hat{B}$). Figure 6 shows the training process of the proposed D-LSTM.

**Figure 7:**
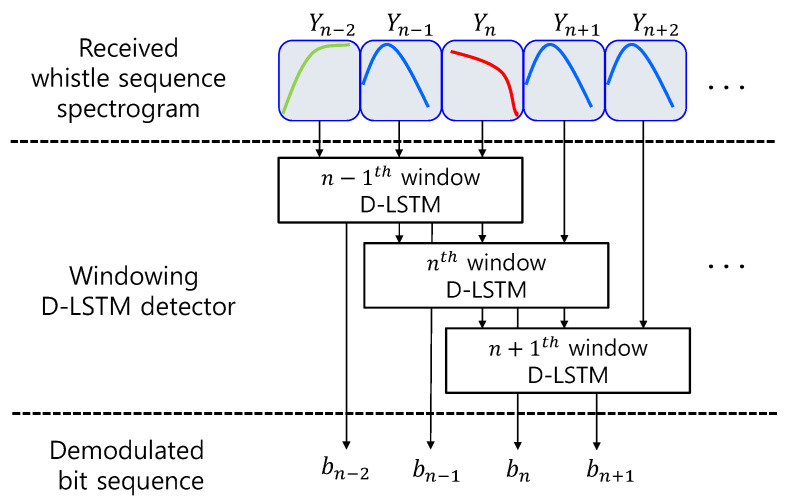
Window processing of D-LSTM.

The length of the transmitted whistle sequence depends on the number of input bits. If the bit length is long and the machine learning networks demodulate all bits, a large memory is needed for the D-LSTM and the computational complexity increases. Thus, the block window is used for the finite memory and the overlapped processing is performed to track the link information. The window size of the proposed D-LSTM is three and the one window span is shifted for overlapping. The window processing procedure is depicted in Figure 7.

In Figure 7, for the processing of the n-th window, the propose D-LSTM receiver detects the bits not only from the n-th window but also from the (n−1)-th windows, and updates the whistle information for the next window. This window processing enables the receiver to use the memory dynamically and reduces the memory size and the computational complexity. The following section mentions the classification and implementation of the processes of the D-LSTM.

## 5. Real Whistle Classification and D-LSTM Implementation

In this section, the classification of the recorded real whistle data is executed, and bit allocation is performed for the reclassified subgroups. Several machine learning networks are also proposed for the implementation.

### 5.1. Whistle Classification

The training whistle data for the proposed biomimetic communication scheme were obtained from Watkins marine mammal sound database. The whistles of white sided dolphin were chosen and the total number of collected whistles was 704 [35]. The feature vectors in Equation (1) were calculated from the 704 whistles and classified into groups using k-means algorithm by Equation (2). The optimal number ($K_{\mathrm{opt}}$) of groups by Equation (2) was eight, and the maximum number ($M_{\mathrm{opt}}$) of bits per one whistle was three.

**Figure 8:**
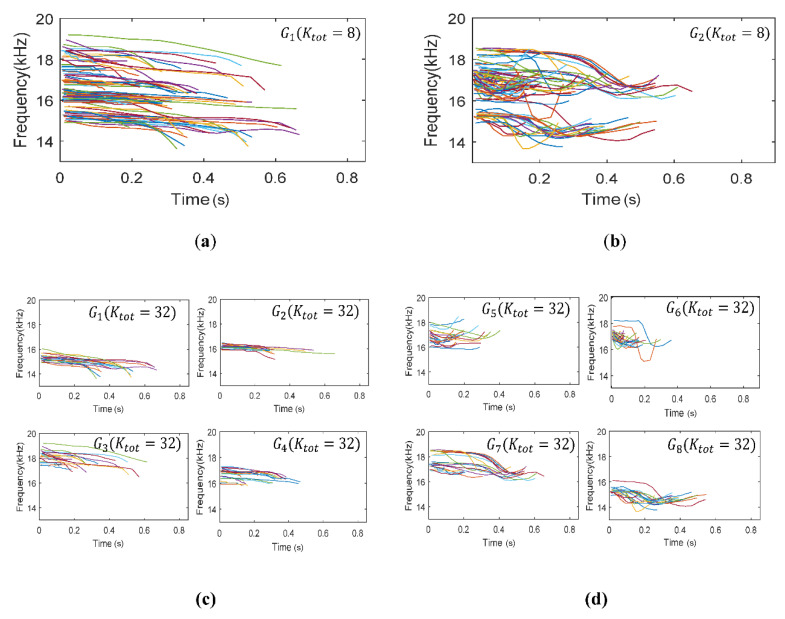
Dolphin whistles classification results; (**a**) $G_{1}(K_{\mathrm{opt}}=8)$; (**b**) $G_{2}(K_{\mathrm{opt}}=8)$; (**c**) $G_{1\sim 4}(K_{\mathrm{tot}}=32)$; (**d**) $G_{4\sim 8}(K_{\mathrm{tot}}=32)$.

For increasing the BER and DoM performances, the eight groups were reclassified into the lager number of subgroups. An expanding factor of α was set as two, and the groups were reclassified into 32 subgroups, i.e., $K_{\mathrm{tot}}=32$. Figure 8 represents some examples of classified 704 whistles by the eight groups and 32 subgroups.

In Figure 8, the horizontal and vertical axes denote the time and the frequency, respectively. Figure 8a,b represents the examples among the classified eight groups by Equation (2), and Figure 8c,d shows the examples of the reclassified 32 subgroups from Figure 8a,b, respectively. Figure 8a,b displays the down chirp patterns and scoop shapes, respectively. The whistles of the subgroups in Figure 8c were separated by the frequency and the time duration which are one of dominant features of the whistles. The whistles of the subgroups in Figure 8d were reclassified by the chirp shape that is another feature of the whistles.

**Figure 9:**
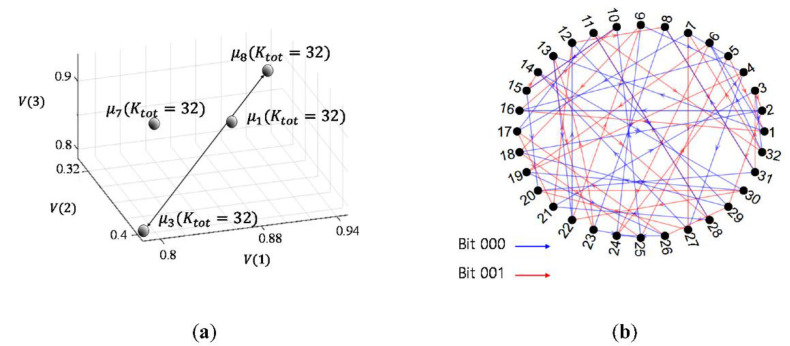
(**a**) $\mu_{1},\mu_{3},\mu_{7},\mu_{8}$ in vector space; (**b**) Adjacent dependency among whistle subgroups.

The element averages, e.g., $\mu_{1},\mu_{3},\mu_{7},\mu_{8}$, of the subgroups from V(1) to V(3) are shown in Figure 9a. In Figure 9a, the distance between $G_{3}$ and $G_{8}$ is the farthest.

The proposed method generates H that presents the link information among subgroups. Multiple H values can be available as described in Section 3. In this example, the number of the subgroup was 32 and the size of one subgroup set was eight. For H, all possible subgroup sets were listed in the order of the largest distance among subgroups, and the short link loop within a few subgroup sets were avoided, and all subgroups were evenly shown in the H matrix. Then, H was made with the size of 32×8. Since the size of H is large, we omit the example of H, but a link example is displayed in Figure 9b. In Figure 9b, the blue line and the red line denote the link information for bits 000 and 001, respectively. For the transmission, as in Section 3, the input bits are mapped into a whistle subgroup indicated by H and one of whistles in the subgroup is randomly chosen and transmitted.

In the next subsection, the structures of the proposed machine learning networks and the learning results are presented.

### 5.2. Implementations of the Proposed Machine Learning Networks

**Figure 10:**
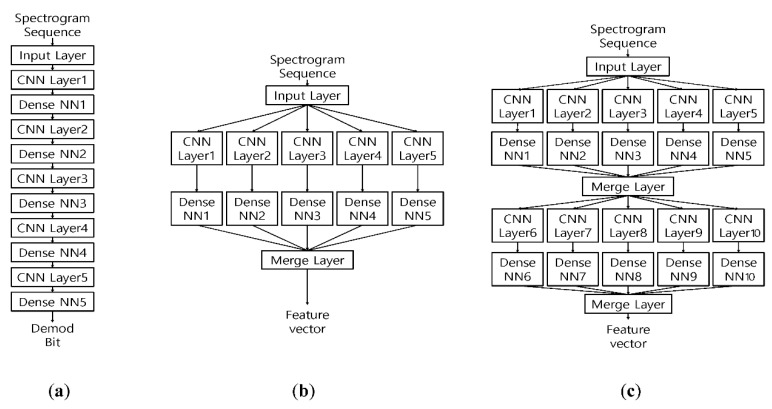
Proposed network architectures (**a**) DNN; (**b**) DAG-net1; (**c**) DAG-net2.

**Figure 11:**
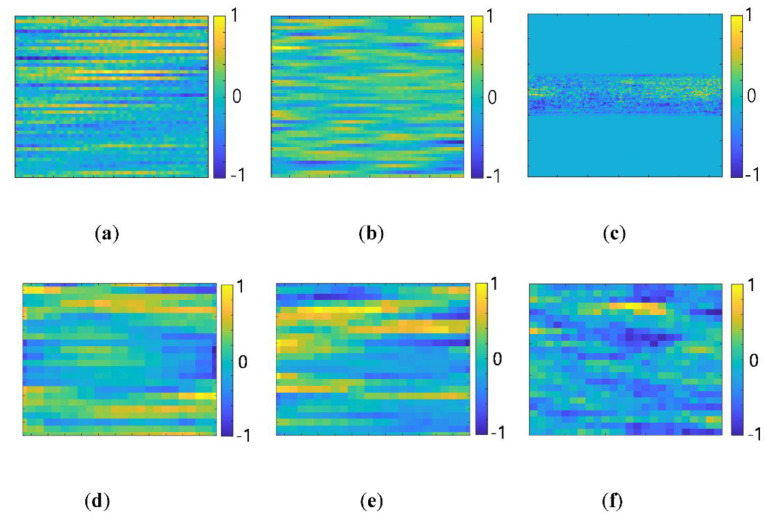
Trained network coefficients (**a**) DNN: Conv layer1 (48 × 48); (**b**) DAG-net1: Conv layer1 (48 × 48); (**c**) DAG-net1: Conv layer1 (128 × 128); (**d**) DNN Conv layer5 (24 × 24); (**e**) DAG-net1: Conv layer5 (24 × 24); (**f**) DAG-net2: Conv layer10 (24 × 24).

The proposed D-LSTMs learnt the spectrograms of the whistles to demodulate the input bits. The spectrum size of the one whistle was set as 65×550. For the whistle feature extraction, we utilized three machine learning networks such as DNN, DAG-net1 and DAG-net2, shown in Figure 10. In Figure 10, the DAG-net2 had two stages of the parallel Convolution Neural Network (CNN)s with merging, while the DAG-net1 had one stage with merging. All CLs of the DNN, DAG-net1, and the second stage of DAG-net2 had the same structure with the same filter size. For the link information, Figure 11b,c are combined with the Bi-LSTM, which are named as D-LSTM1 and D-LSTM2, as in Figure 5. The D-LSTM1 and the D-LSTM2 were developed since the conventional DNN and DAG-net does not attain a great enough BER performance.

The performances of three structures are compared in Section 6. The DAG-net1 and the DAG-net2 outperformed the conventional DNN, and the DAG-net2 showed a better performance than the DAG-net1. As the number of stages of the DAG-net increases, the performance of networks also increases. Figure 11 demonstrates some examples of filter coefficients in the CLs of each network when the training is done. In Figure 11, all networks show some concentrated values of the filter coefficient dimension.

For all networks, when the layer number increased, the large value coefficients tended to converge to some specific regions. Among three methods, DAG-net2 demonstrated highly concentrated values at a certain spot, while the DNN and the DAG-net1 showed spread coefficients. This observation means that the DAG-net2 seems to effectively come up with the features of the whistle patterns.

In the next section, the computer simulations and practical ocean experiment results are shown for the communication performance comparisons of the proposed biomimetic methods with the conventional methods.

## 6. Simulation and Ocean Experiments

This paper proposes a biomimetic modulation method using real dolphin whistles with link information and a detection scheme using the D-LSTM for the UWA channel distorted whistles. The performance comparisons were focused on two aspects: the first one is the whistle-by-whistle detection capability of the proposed machine learning networks. For the whistle-by-whistle detection comparisons, the BERs of the DAG-net1 and the DAG-net2, and the conventional ML detection, and the conventional DNN were compared. The second one is the detection performance by the additional link information. The BER performances of the D-LSTM1 and the D-LSTM2 were compared with those of the DAG-net1 and the DAG-net2. These comparisons were tested in computer simulations and practical ocean experiments.

The communication parameters used for the comparisons were $M_{\mathrm{opt}}=3$, $K_{\mathrm{opt}}=8$, α=2, $K_{\mathrm{tot}}=32$ which are the same as in Section 5. For fair comparisons, the same DoM was kept for all algorithms, i.e., all 704 whistles were utilized for the transmissions. The learning processes of all algorithms were executed for one million whistle spectrograms in the computer simulation and the learning results were also used for the practical ocean experiments.

### 6.1. Simulation Result

**Figure 12:**
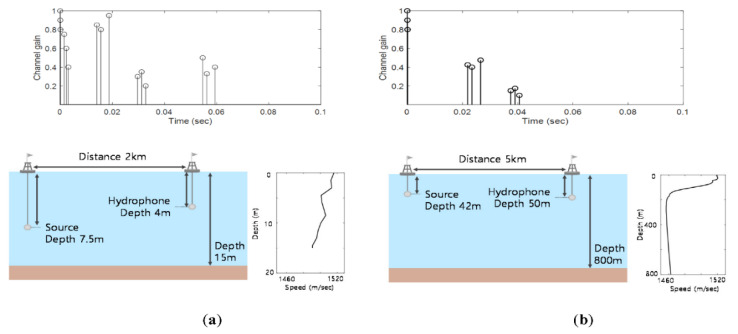
UWA channel (**a**) for training; (**b**) for tests.

For the learning process, the whistles with the Additive White Gaussian Noise (AWGN) channel and with the time varying multipath channel of a shallow water in Figure 12a were utilized. The simulation models of the ocean depths, the distance between the transmitter and the receiver, and the depth of each transmission source and receiver hydrophone are also shown in Figure 12a. For the UWA channel, SNRs vary from −16 dB to 14 dB by a 2 dB step, and 150 cases were generated for a SNR to obtain statistical reliability. At every iteration, Doppler frequencies of each multipath randomly and independently varied from 0 Hz to 2 Hz. These procedures were executed for all 704 whistles. Thus, 3.4 million training samples were utilized to train the D-LSTM network. Please note that the UWA channel had long multi-path delay times, which covered other conventional UWA channels. The efficacy of this learning process was proven in computer simulations and ocean experiments. For the over-fitting problem of the machine learning performance test, the different ocean environments with different multipath channels, given in Figure 12b, were also used.

**Figure 13:**
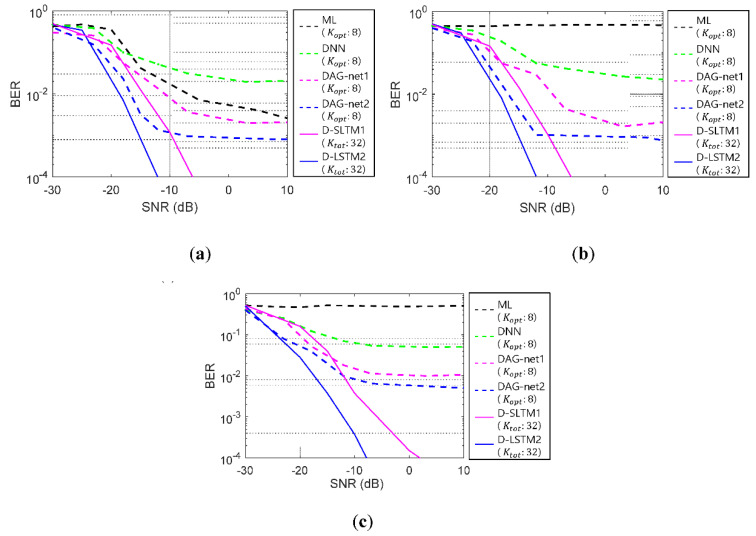
Simulation bit error rate (BER) results (**a**) AWGN; (**b**) learning UWA channel; (**c**) test UWA channel. ML: maximum likelihood.

In Figure 13, the BER performances of the tested algorithms are displayed. The dashed lines denote the BERs of whistle-by-whistle detections. The solid lines denote the BERs of the proposed link information aided detection methods. The black, the green, the pink, and the blue dashed lines denote the BERs of the conventional ML detectors, the conventional DNN, DAG-net1, and DAG-net2, respectively. The pink and the blue solid lines denote the BERs of the D-LSTM1, and the D-LSTM2, respectively. Figure 13a–c exhibits the BERs of the AWGN channel, the UWA channel of Figure 12a, and the different UWA channel of Figure 12b, respectively.

In Figure 13, all BER results of the dashed lines exhibited the error flows by the limitation of the whistle-by-whistle detection. The error-floors started with 10^−2^, which were considered as a low value in the conventional image detection applications. These values, however, may not be acceptable for the communications. Among the tested whistle-by-whistle detectors, the DAG-net1 and the DAG-net2 showed better BER performances than the other conventional algorithms, and had error-floors at 10^−3^. As the number of the DAG-net stages increased, the detection performance also increased. Thus, the proposed D-LSTM1 and D-LSTM2 were tested for detection of the link information. In Figure 13, the proposed D-LSTM2 method demonstrated better BER performance than other algorithms, and did not have the error-floor. In Figure 13c, the BER results were obtained by the UWA channel in Figure 12b, which was not used for the training. Even though the trainings were executed by AWGN and the UWA channel in Figure 12a, the BER results of the proposed method for the different UWA channel in Figure 12b, also demonstrated the lowest BER value. Please note that the multipath of the UWA channel severely distorts the transmitted signals and the maximum delay time of the multipath is one of the important parameters of the UWA communications. The multipath delay time of the trained UWA channel in Figure 12a was larger than that of Figure 12b. Therefore, the proposed algorithm was expected to attain the good BER results for practical ocean experiments. In the next subsection, the BERs of the practical ocean experiments are demonstrated.

### 6.2. Ocean Experiments

**Figure 14:**
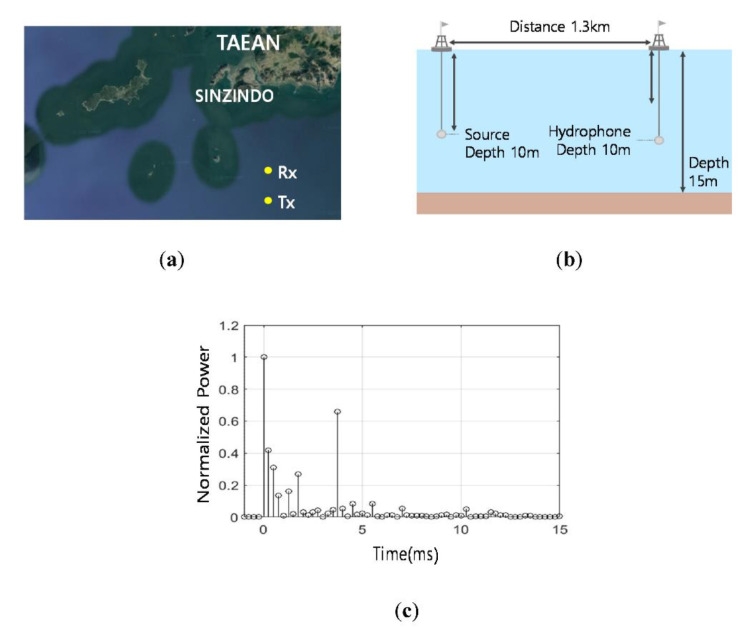
Ocean experiments environments (**a**) Location; (**b**) Configuration; (**c**) Channel delay profile.

The practical ocean experiments were performed for evaluating the BER performance of the proposed method based on the D-LSTM and the conventional CNN based method. The learning process of the proposed D-LSTM was the same as the simulation subsection, which was different from the practical ocean channel. For the ocean experiments, the location and depths and the delay profile of the transmitter and the receiver are shown in Figure 14. The transducer was Neptune-D17B with a bandwidth from 12.5 kHz to 19.5 kHz, and the hydrophone was TC4032. The location of the ocean experiments was at a point in the west Sea of South Korea, which was 4.2 km apart from Sinzindo. In Figure 14c, the UWA channel of the practical ocean is shown, and the UWA channel was estimated by the Linear Frequency Modulation (LFM)-chirp that was attached before the data transmission only for the observation of the UWA channel. Note that the LFM-chirp was not used when the proposed signal was demodulated, and the practical ocean UWA channel is different from those of the simulations in Figure 12a,b.

**Figure 15:**
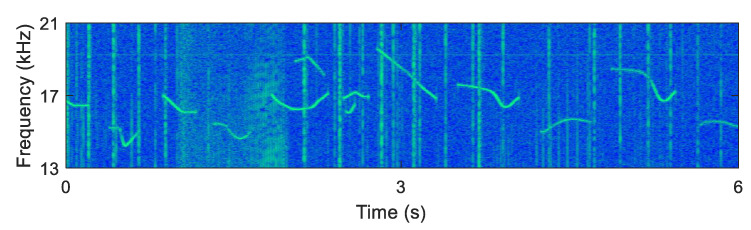
The spectrogram example of the received signals in the ocean experiments.

The parameters of the transmission modulator and the machine learning networks used for the ocean experiments were the same as those of computer simulations. The spectrogram examples of the received signal in the practical ocean experiments are displayed in Figure 15.

For the BER performance calculation, 5000 whistles were transmitted, i.e., 15,000 bits were transmitted. The BER results of the ocean experiment are calculated in Table 2.

In Table 2, the BERs of the whistle-by-whistle detection methods of the ML and the DNN are shown to be 0.36 and 0.37, respectively, which were large and not useful to the practical communications. The DAG-net1 and the DAG-net2 displayed better BER performances than the ML and the DNN, but the BERs of the DAG-nets themselves are still large. However, the proposed D-LSTM1 and D-LSTM2 that utilized the link information, demonstrated lower BER values than other algorithms. These results were well matched with those of the computer simulations. In addition, the fact that the proposed learning process is enough to obtain a low BER without learning of the practical ocean UWA channel is proven.

Therefore, the proposed D-LSTM2 showed the best BER performance compared to other algorithms, and the whistle-by-whistle detection scheme was not used for the whistle based biomimetic communications, and the additional link information played an important role in increasing the BER performance. In addition, the proposed algorithm utilizes all whistles and preserves the maximum DoM, which is crucial to the UWA covert communications.

## 7. Conclusions

In this paper, we propose a machine learning based biomimetic covert acoustic communication method that mimics dolphin whistles without whistle distortion and preserves the maximum DoM with a low BER. For the transmission, the proposed method modulates the whistle itself for the bit allocation and provides the link information among consecutive whistles. For the receiver, the proposed method utilizes the D-LSTM that extracts the whistle features and detects the bits using the link information. Computer simulations and the practical ocean experiments were performed for the BER comparisons of the proposed algorithm with those of the other conventional detection algorithms. The BER performance of the proposed D-LSTM2 outperforms other conventional detection methods in both computer simulations and the practical ocean experiments.

Many dolphins live together and communicate at the same time. Thus, for the future work, the transmission and the detection of multiple dolphin whistles needs to be developed to obtain a larger DoM and to increase the data rate.

## Figures and Tables

**Table 1:** List of bio-mimetic communication schemes.

Author	Title	Year
Han. X. et al.	Covert underwater acoustic communication using dolphin sounds [8]	2013
Liu. S. et al.	Bionic communication by dolphin whistle with continuous-phase based on Minimum Shift Keying (MSK) modulation [9]	2016
Liu. S. et al.	Biologically inspired covert underwater acoustic communication by mimicking dolphin whistles [10]	2017
Jian. J. et al.	Bio-inspired steganography for secure underwater acoustic communication [11]	2018
Ahn. J. M. et al.	Multipath combining method for frequency shift keying underwater communications mimicking dolphin whistle [12]	2018
Ahn. J. M. et al.	Mimicking dolphin whistles with continuously varying carrier frequency modulation for covert communication [13]	2019
Ahn. J. M. et al.	Machine learning based dolphin whistle transceiver for bio-inspired underwater covert communication [14]	2019
Lee. H. J. et al.	Time-frequency modulation based mimicking dolphin whistle for covert underwater acoustic communication [15]	2020

**Table 2:** BERs of the ocean experiments.

Detection Schemes	ML	DNN	DAG-net1	DAG-net2
Whistle-by-whistle detection ($K_{\mathrm{opt}}=8$)	0.37	0.36	0.09	0.046
With additional link information ($K_{\mathrm{tot}}=32$)	-	-	0.012	0.002
